# Effects of Yogurt Intake on Cardiovascular Strain during Outdoor Interval Walking Training by Older People in Midsummer: A Randomized Controlled Study

**DOI:** 10.3390/ijerph19084715

**Published:** 2022-04-13

**Authors:** Koji Uchida, Ryutaro Shimamura, Ryo Ikefuchi, Mayuko Morikawa, Mayuka Furihata, Masaaki Hanaoka, Hiroshi Nose, Shizue Masuki

**Affiliations:** 1 Department of Sports Medical Sciences, Shinshu University Graduate School of Medicine, Matsumoto 390-8621, Japan; uchidakoji@shinshu-u.ac.jp (K.U.); 20ms006a@shinshu-u.ac.jp (R.S.); 20ms002h@shinshu-u.ac.jp (R.I.); m_mori@shinshu-u.ac.jp (M.M.); mayuka@shinshu-u.ac.jp (M.F.); 2 Department of e-Health Sciences, Shinshu University Graduate School of Medicine, Matsumoto 390-8621, Japan; mhanaoka@shinshu-u.ac.jp (M.H.); nosehir@shinshu-u.ac.jp (H.N.); 3 Institute for Biomedical Sciences, Shinshu University, Matsumoto 390-8621, Japan; 4 Jukunen Taiikudaigaku Research Center, Matsumoto 390-8621, Japan

**Keywords:** interval walking training, exercise intensity, heart rate, dairy supplementation, IoT

## Abstract

We examined whether post-exercise yogurt intake reduced cardiovascular strain during outdoor interval walking training (IWT) in older people during midsummer. The IWT is a training regimen repeating slow and fast walking at ~40% and ≥70% peak aerobic capacity, respectively, for 3 min each per set, ≥5 sets per day, and ≥4 days/wk. We randomly divided 28 male and 75 female older people (~73 yr), who had performed IWT ≥12 months, into a carbohydrate group (CHO-G) consuming jelly (45 g CHO, 180 kcal) and a yogurt group (YGT-G) consuming a yogurt drink (9.3 g protein, 39 g CHO, 192 kcal) immediately after daily IWT for 56 days while monitoring exercise intensity and heart rate (HR) with portable devices. We analyzed the results in 39 subjects for the CHO-G and 37 subjects for the YGT-G who performed IWT ≥ 4 days/wk, ≥60 min total fast walking/wk, and ≥4 sets of each walk/day. We found that the mean HR for fast walking decreased significantly from the baseline after the 30th day in the YGT-G (*p* < 0.03), but not in the CHO-G (*p* = 1.00). There were no significant differences in training achievements between the groups. Thus, post-exercise yogurt intake might reduce cardiovascular strain during outdoor walking training in older people.

## 1. Introduction

The incidence of heat illness during midsummer has rapidly increased in the past several decades as the ambient temperature (T_a_) has increased globally [1]. This tendency has been more prominent in the elderly than in young adults who are more tolerant to heat stress [2,3]. To prevent the development of heat-related illness, aerobic exercise training above a given intensity has been recommended [4,5]. In addition, nutritional supplementation during the training has been reported to enhance the effects [6,7]. However, these results were obtained by means of laboratory- or gym-based studies which were designed to ensure exercise intensity (EI) and volume during training, so it has remained unknown whether the same effects could be obtained in actual field studies.

Previously, we have suggested that post-exercise milk protein-carbohydrate (CHO) supplementation during aerobic training for a given period can increase plasma volume (PV) and thermoregulatory capacity, more than placebo supplementation in laboratory- and gym-based studies, with a tendency toward an increase in aerobic capacity in older people [6,7]. However, studies of this nature typically require machines for training, treadmill or bicycle ergometers, and institutions where the machines are installed, as well as staff who instruct subjects to perform training at a given EI to acquire the effects. Thus, such studies involve considerable institutional and personnel resources, which naturally limits the number of subjects and the amount of time available for evaluating the effects.

To solve these problems, we developed the e-Health Promotion System (Kissei Comtec, Matsumoto, Japan), composed of interval walking training (IWT), a portable calorimeter (JD Mate; Kissei Comtec, Matsumoto, Japan), and an Internet of things (IoT) system for use by middle-aged and older people to perform IWT at their favorite time and place, without going to any specific institutions [8]. IWT is a training regimen repeating ≥ 5 sets of alternative slow and fast walking at ~40% and ≥70% peak aerobic capacity ($\overset{\cdot }{V}$O_2peak_) for 3 min each per set for ≥4 days/wk. The portable calorimeter is a device equipped with a tri-axial accelerometer and a barometer, which is used to measure EI every min during IWT as precisely as respiratory gas analysis [9]. The IoT system was developed for accumulating training achievements and the effects of IWT on physical fitness and lifestyle-related disease symptoms [8]. Through our work with this system, we have demonstrated that the high-intensity walking time, rather than the low-intensity walking time, during IWT is a key determinant to increase physical fitness and improve health outcomes in middle-aged and older people [10]. In the present study, we used the system to confirm that EI and volume during IWT reached the levels which have been suggested to improve aerobic and thermoregulatory capacities in laboratory- or gym-based studies [6,7].

With this approach in the present study, we sought to examine the effects of milk protein-CHO supplementation during outdoor IWT on aerobic and thermoregulatory capacities. To evaluate time-series changes in these capacities over the intervention period, we measured heart rate (HR) every sec with a portable HR meter (M200, Polar, Kempele, Finland) during daily IWT. The reason for adopting HR as a combined index for aerobic and thermoregulatory capacities was that HR at a given EI decreases as $\overset{\cdot }{V}$O_2peak_ increases, increasing during a constant intensity of exercise as body core temperature increases, a phenomenon known as “cardiovascular drift” [6,11]. Therefore, we supposed that if aerobic and thermoregulatory capacities increased along with training, it would reduce the increase in HR at a given EI during outdoor IWT.

For this study, we used a non-fat yogurt drink for supplementation, since it contains amounts of milk protein and CHO similar to those of the supplements used in the previous laboratory- and gym-based studies, and since it is commercially available in bottle form [6,7]. In addition, yogurt, which is highly popular in Japan, is reported to induce less diarrhea incidence by accelerating intestinal lactose absorption when compared with the effects of plain milk on older people in Japan [12,13].

Thus, the continuous and simultaneous measurements of HR and EI at every training session throughout an intervention period in a large number of older people would enable us to detect any merits of the post-exercise yogurt supplementation in the field, which had never before been detected due to low statistical power caused by limited timing of the measurements and by a limited number of subjects. In the present study, we hypothesized that HR at a given EI during outdoor IWT in midsummer would be reduced by the yogurt supplementation more than by the placebo supplementation in older people.

## 2. Methods

### 2.1. Subjects and Grouping

**Figure 1:**
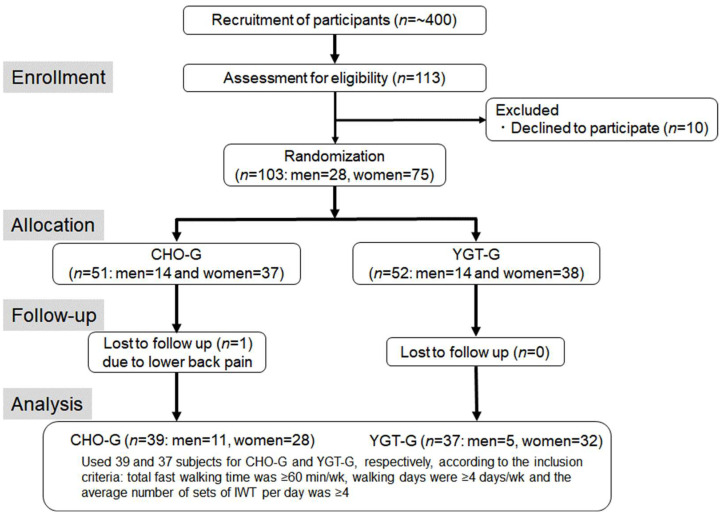
CONSORT flow diagram. CHO-G, the carbohydrate intake group; YGT-G, the yogurt intake group.

Figure 1 shows a timeline of the present study. The subjects were recruited from participants who had performed IWT for more than 12 months in the “Jukunen Taiikudagaku” program, a long-running health promotion program for middle-aged and older people in Matsumoto City, Japan. The reason for recruiting subjects who had performed IWT before was that the effects of IWT, such as increased aerobic capacity and improved lifestyle-related disease symptoms, were observed for the first six months of the training, but thereafter, the effects reached plateau levels [14]. Accordingly, we believed that it would enable us to detect the mere merits of the supplementation by excluding any effects of the training by itself. For recruitment, we displayed a poster in a local community office that the participants visit regularly, and we also distributed leaflets. After the experimental protocol was fully explained to ~400 participants, 103 of 113 responders (28 men and 75 women; age, 60–83 years) provided written informed consent and enrolled in the study. Then they were randomly divided into two groups according to $\overset{\cdot }{V}$O_2peak_: a yogurt group (YGT-G, 14 males and 38 females) consuming a non-fat yogurt, and a carbohydrate group (CHO-G, 14 males and 37 females), consuming a CHO solution within 30 min after daily IWT. For the grouping, we used $\overset{\cdot }{V}$O_2peak_ measured in October 2019, the last value of the measurements which we had determined regularly every six months to ensure individual target intensity for fast walking of IWT for all participants in the program, since it was prohibited by the government to gather a group of subjects in a gym during the subsequent COVID-19 pandemic. However, we were able to confirm that $\overset{\cdot }{V}$O_2peak_ increased sharply for the first six months after starting IWT, but reached a plateau thereafter, even while the participants continued training [14]. The physical characteristics and the past and current health status before training in subjects adopted for analyses (see below) are shown in Table 1 and Table 2, respectively.

Given the nature of the supplements, subjects and investigators were not blinded to the grouping, but the subjects did not know which supplement was expected to be more effective than the other.

### 2.2. Protocol

For IWT, the subjects in both groups were instructed to continue to repeat ≥5 sets of fast (≥70% $\overset{\cdot }{V}$O_2peak_) and slow (~40% $\overset{\cdot }{V}$O_2peak_) walking for 3 min each per set, ≥4 days per week, as they had done prior to participating in the present study. The subjects in YGT-G were instructed to consume a yogurt drink, while those in CHO-G consumed an energy jelly within 30 min after daily IWT, respectively. The nutritional components of the respective supplements are described below. The subjects in both groups were also instructed not to consume any other foods or fluids, except for either water or barley tea, with no caffeine or sugar, during IWT and for 60 min before and after IWT. The intervention period was 56 days from 27 July to 20 September 2020, where T_a_ and relative humidity (RH) on average, were 25.6 ± 2.6 (SD) °C and 68 ± 10% at 9:00 and 28.2 ± 3.1 °C and 59 ± 11% at 17:00, the two times of day around which the subjects performed IWT most frequently. There was no harmful event during the intervention. The training achievements during the intervention in the subjects adopted for analyses (see below) are shown in Table 3.

### 2.3. Supplements

The nutritional composition of the yogurt drink for YGT-G and that of the jelly drink for CHO-G are shown in Table 4.

### 2.4. Measurements

#### 2.4.1. $\overset{\cdot }{V}$O_2peak_

$\overset{\cdot }{V}$O_2peak_ was determined by measuring energy expenditure every 5 s with the calorimeter (JD-Mate) carried on the midclavicular line of the right or left front waist during graded-intensity walking on a flat floor at subjective slow, moderate, and fast speeds for 3 min each, as reported previously [15]. Regarding the precision, $\overset{\cdot }{V}$O_2peak_ determined by the graded walking exercise was highly correlated with that obtained by graded cycling exercise with respiratory gas analysis (R^2^ = 0.83, *p* < 0.0001), and furthermore, the regression coefficient was close to a unit in middle-aged and older men and women (*n* = 278) and ±0.23 L/min of the 95% confidence limit over the range of $\overset{\cdot }{V}$O_2peak_ variation [8,15]. Thus, the $\overset{\cdot }{V}$O_2peak_ determined by the graded walking test was reliable enough to be used for the grouping.

#### 2.4.2. EI during IWT

EI and volume during the training period were monitored with the portable calorimeter (JD-Mate), similar to the $\overset{\cdot }{V}$O_2peak_ measurement, but recorded every minute. We confirmed that energy expenditure every min measured with the meter was in good agreement with the value simultaneously measured by respiratory gas analysis during walking in an outdoor course including inclines [9]. A beeping signal alerted the subjects at the specified timing to change the intensity, and another melody announced to them when the intensity had reached the target level every min. The subjects visited a local community office once a month to have their walking record transferred from the meter to a central server at the administrative center through the internet for automatic analysis and reporting by the e-Health Promotion System.

#### 2.4.3. HR

A wrist band type of HR monitor (M200; Polar) was used to record HR every second during IWT. The recorded data were transferred to a cloud-based internet server (Polar Flow, https://account.polar.com, accessed on 12 April 2022) when subjects visited the local community office once a month.

#### 2.4.4. Dietary Intake

All subjects were instructed to maintain their dietary habits during the intervention period. In addition, subjects were instructed to report the foods for the 4th and 8th week of the intervention by answering a questionnaire that was prepared by a dietician (FFQg Ver 3.5; Kenpakusha, Tokyo, Japan). The average values of each nutritional composition are shown in Table 5.

## 3. Analyses and Statistics

As shown in Figure 1, one subject in the CHO-G dropped out due to lower back pain and was excluded from analyses. To analyze the HR response to a given EI during IWT, we adopted 37 (5 men and 32 females) of 52 subjects in the YGT-G and 39 (11 men and 28 females) of 51 subjects in the CHO-G using the following 3 criteria: (1) the average total fast walking time per week was ≥60 min, (2) the walking days were ≥4 days per week, and (3) the average sets of slow and fast walking per day were ≥4. The first 2 criteria were applied to secure the training volume above a given intensity to increase aerobic [8,10,15] and thermoregulatory capacities suggested in the laboratory-based studies [6,7], and the third criterion was adopted to compare HR at a given EI between the first and the last sets of IWT for each day.

**Figure 2:**
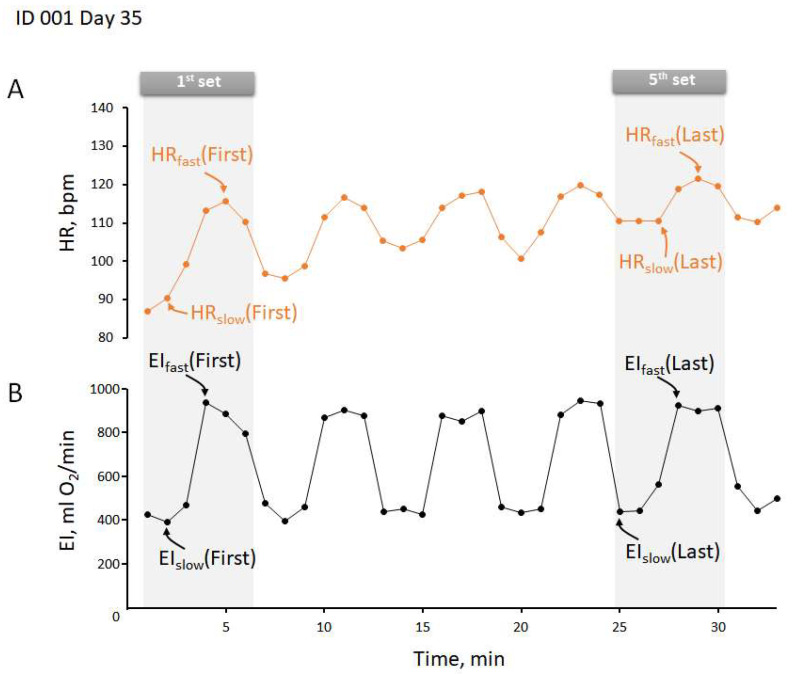
A typical example of heart rate (HR) (**A**) and exercise intensity (EI) (**B**) responses during IWT. HR_slow_ and HR_fast_ are HR for slow and fast walking, respectively. Similarly, EI_slow_ and EI_fast_ are EI for slow and fast walking. The suffixes of “First” and “Last” indicate HR and EI for the first set and the last set of IWT, respectively.

Figure 2 shows a typical example of EI and HR responses during five sets of slow and fast walking during IWT for a day in one subject. As shown in the figure, the EIs during slow and fast walking remained almost unchanged, while the HRs during each walking speed gradually increased along with the walking. Therefore, we assumed that HR during the first set of IWT was determined mainly by EI relative to individual aerobic capacity, with minimal influence of increased body temperature, and the gradual increase in HR along with the walking reflected the increasing rate of body temperature [16]. Accordingly, we determined the lowest values of EI and HR after the 1st min of slow walking and their highest values after the 1st min of fast walking for the first set of IWT as an index of aerobic capacity, while considering the delayed response time of the HR monitor after changing walking speed determined in a preliminary study, and the respective EI and HR values for the last set of IWT as an index of aerobic and thermoregulatory capacities. In addition, we determined the differences in EI (ΔEI) and HR (ΔHR) between the first and last sets of IWT in each day as an index of thermoregulatory capacity. These determinations were completed on each day of the training period in all subjects to examine any time-series changes of aerobic and thermoregulatory capacities along with the training.

To assess the profiles of EIs and HRs and those of ΔEI and ΔHR for each day during the training for 56 days, we divided the intervention period into 10 bins consisting of 5 days, plus an 11th bin of 6 days, and determined the mean bottom values of EI and HR during slow walking and their peak values during fast walking for each bin. When there was a no walking day for a bin for a subject, the value was copied from that in the preceding bin by the last observation carried forward method [17].

To adjust the time axis between the EI and HR files, which were separately recorded every minute and every second, respectively, with different portable devices and downloaded from the different servers, we used cross-correlation analysis between them. For this analysis, the HR recorded every second was averaged for a period from *t* − 30 to *t* + 30 s, while moving *t* by an increment of 1 s so that the cross-correlation function with EI marked the maximal values.

**Figure 3:**
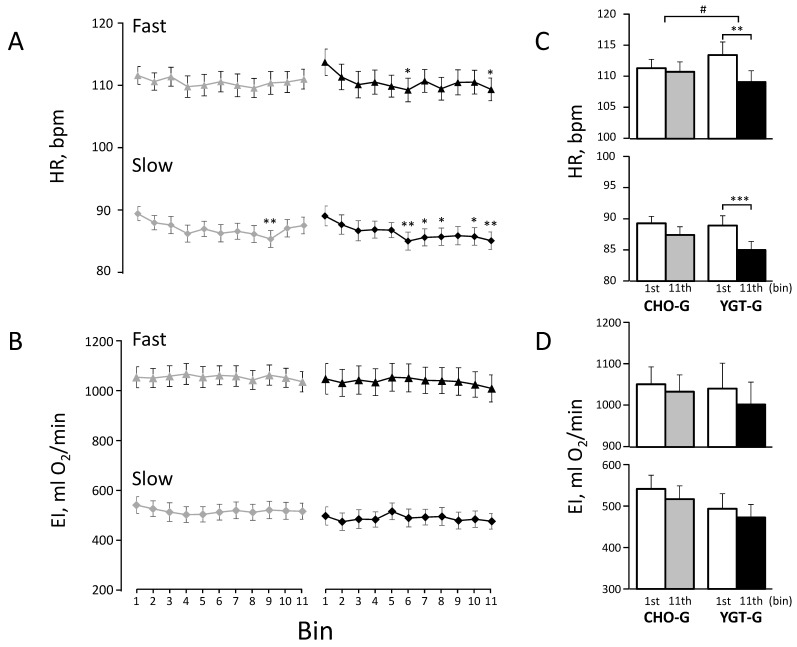
The profiles of heart rate (HR) (**A**) and exercise intensity (EI) (**B**) for the first set of IWT throughout the intervention in both groups. The gray lines and symbols indicate the values in the carbohydrate intake group (CHO-G), and the black lines and symbols indicate those in the yogurt intake group (YGT-G). The comparison of HR_fast_ and HR_slow_ between the 1st and 11th bins are presented in the upper and lower panels, respectively (**C**). Similarly, the comparison of EI_fast_ and EI_slow_ between the 1st and 11th bins are presented in the upper and lower panels, respectively (**D**). The open columns indicate HR or EI in the 1st bin for both groups, and the gray and the black columns indicate HR or EI in the 11th bin for CHO-G and YGT-G, respectively. Values are presented as means ±SE bars for 39 subjects in CHO-G and for 37 subjects in YGT-G. *, **, and *** indicate significant differences vs. the 1st bin at the levels of *p* < 0.05, *p* < 0.01, and *p* < 0.001, respectively. # indicates significant interactive effect, bins (the 1st vs. the 11th bins) x groups (CHO-G vs. YGT-G), at the level of *p* < 0.05.

**Figure 4:**
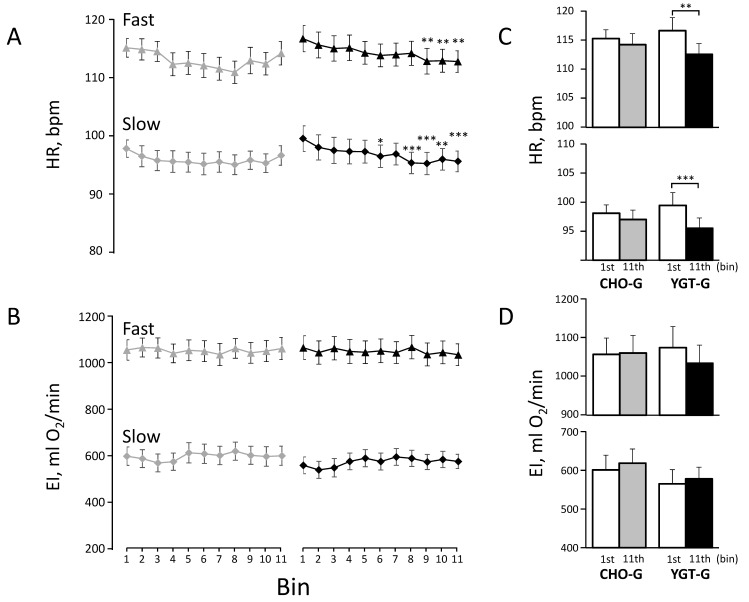
The profiles of heart rate (HR) (**A**) and exercise intensity (EI) (**B**) for the last set of IWT throughout the intervention in both groups. The comparison of HR_fast_ and HR_slow_ between the 1st and 11th bins are presented in the upper and lower panels, respectively (**C**). Similarly, the comparison of EI_fast_ and EI_slow_ between the 1st and 11th bins are presented in the upper and lower panels, respectively (**D**). *, **, and *** indicate significant differences vs. the 1st bin at the levels of *p* < 0.05, *p* < 0.01, and *p* < 0.001, respectively. Other symbols and abbreviation are the same as in Figure 3.

**Figure 5:**
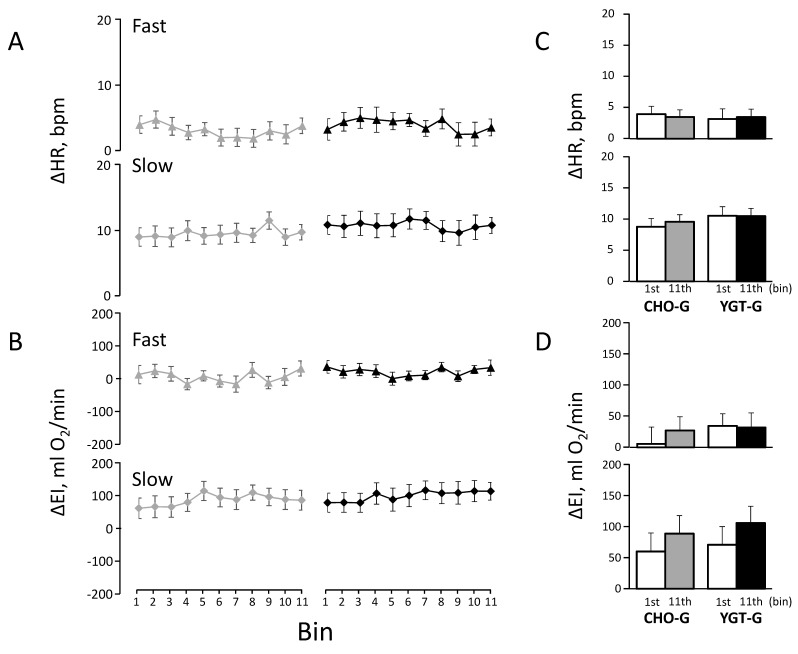
The profiles of the differences in heart rate (ΔHR) (**A**) and exercise intensity (ΔEI) (**B**) between the first and last sets of IWT throughout the intervention in both groups. The comparison of ΔHR_fast_ and ΔHR_slow_ between the 1st and 11th bins are presented in the upper and lower panels, respectively (**C**). Similarly, the comparison of ΔEI_fast_ and ΔEI_slow_ between the 1st and 11th bins are presented in the upper and lower panels, respectively (**D**). The symbols and other abbreviation are the same as in Figure 3.

A Pearson’s chi-square test was used to examine any significant differences in gender distribution between CHO-G vs. YGT-G. A Kruskal–Wallis test was used to examine any significant differences in the past and current health status between groups (Table 2). A one-way analysis of variance (ANOVA) for the groups (CHO-G vs. YGT-G) was used to examine any significant differences in the physical characteristics (Table 1), training achievements (Table 3), and dietary intake (Table 5) between groups. A one-way ANOVA (1–11 bins) for repeated measures was used to examine any significant differences in EI and HR (Figure 3A,B and Figure 4A,B) and in ΔEI and ΔHR (Figure 5A,B), along with the training in each group. A two-way ANOVA, one-between groups (YGT-G vs. CHO-G) and one-within bins (the 1st vs. the 11th bins), for repeated measures was used to examine any significant differences in EI and HR (Figure 3C,D and Figure 4C,D) and in ΔEI and ΔHR (Figure 5C,D) between the 1st and the 11th bins. A Bonferroni test was used in subsequent post-hoc tests for any pairwise comparisons after confirming significant differences by ANOVA; *p* values < 0.05 were considered significant. Values are expressed as means ± standard error (SE) unless otherwise indicated.

## 4. Results

As shown in Table 1, Table 2, Table 3 and Table 5, there were no significant differences in the physical characteristics, past and current health status, training achievements, and dietary intake during training between CHO-G and YGT-G, respectively. We confirmed no significant difference in gender distribution of the subjects for the analyses between the groups (*p* > 0.1).

The profiles of mean HRs and EIs in each bin during slow and fast walking for the first set of IWT are shown in Figure 3A,B, respectively, and the comparisons between the 1st and 11th bins are presented in Figure 3C,D, respectively, for both CHO-G and YGT-G. As shown in the panels, the EIs for each walking speed in both groups remained unchanged throughout the training period (Figure 3B), with no significant differences between the 1st and 11th bins (Figure 3D) (*p* > 0.2), yet we found that the HRs during slow and fast walking in YGT-G decreased the most after the 6th bin, compared to the values for the 1st bin (*p* < 0.04 and *p* < 0.03, respectively), while the decreases in HRs for CHO-G were minimal (*p* < 0.01 for slow walking and *p* = 1.00 for fast walking) (Figure 3A). As a result, we confirmed significant differences in the HRs between the 1st and 11th bins in YGT-G (both, *p* < 0.003) but not in CHO-G (*p* > 0.08) (Figure 3C), with a significant interactive effect of two-way ANOVA—bins (the 1st vs. the 11th bins) x groups (CHO-G vs. YGT-G)—on HR during fast walking (*p* = 0.033).

Similar to Figure 3, Figure 4 shows the profiles of mean HRs (Figure 4A) and EIs (Figure 4B) in each bin for the last set of IWT, and the comparisons between the values between the 1st and 11th bins are presented in Figure 4C,D.

Figure 5 shows the profiles of ΔHRs (Figure 5A) and ΔEIs (Figure 5B) during slow and fast walking in each bin of the training, with comparisons between the 1st and 11th bins (Figure 5C,D) for CHO-G and YGT-G. As shown in the figures, there were no significant differences in the profiles of ΔHR and ΔEI during slow and fast walking between CHO-G and YGT-G (all, *p* > 0.4). Moreover, there were no significant differences in ΔHR and ΔEI between the 1st and 11th bins in both groups (both, *p* > 0.1).

There was no evidence to suggest that the effect of yogurt intake was influenced by gender (Figure 3, Figure 4 and Figure 5) (all, *p* > 0.1).

## 5. Discussion

This is the first study to examine the effects of yogurt intake on cardiovascular strain induced by outdoor IWT in ~80 older subjects, also including many more subjects than the ~20 subjects in the previous laboratory- and gym-based studies [6,7]. In our study, we found that HRs during slow and fast walking for IWT gradually decreased after the 30th day (the 6th bin) of the 56-day training in the YGT-G, while the decreases were minimal in the CHO-G, where we confirmed that EIs for each walking speed were constant during the training in both groups.

### 5.1. Physical Characteristics of Subjects

The effects of the supplementation might depend on the subjects’ baseline physical fitness. Table 1 shows the physical characteristics of the subjects. Regarding their physical fitness levels, $\overset{\cdot }{V}$O_2peak_ was 26.5 and 25.1 mL·kg^−1^·min^−1^ for CHO-G and YGT-G, respectively, on average. In a previous study [18], we divided 468 women aged ~64 yr equally into 3 groups (low, middle, and high) according to their $\overset{\cdot }{V}$O_2peak_, and reported that $\overset{\cdot }{V}$O_2peak_, on average, was 17.2, 21.5, and 25.9 mL·kg^−1^·min^−1^, respectively, for the three groups. Similarly, we divided 198 men aged ~68 yr equally into 3 groups (low, middle, and high) and reported that $\overset{\cdot }{V}$O_2peak_, on average, was 16.3, 20.0, and 25.4 mL·kg^−1^·min^−1^, respectively. In addition, when we compared the baseline $\overset{\cdot }{V}$O_2peak_ value in the present study with the values in the past laboratory- and gym-based studies where we had examined the effects of milk protein-CHO supplementation during training [6,7], it was lower than ~35 mL·kg^−1^·min^−1^ in male subjects aged ~67 yr [6], but similar to ~28 mL·kg^−1^·min^−1^ in male subjects aged ~69 yr [7]. Thus, the subjects in the present study belonged to the high $\overset{\cdot }{V}$O_2peak_ group of this age, and their $\overset{\cdot }{V}$O_2peak_ was within the range of that of subjects in the laboratory-based studies [6,7].

### 5.2. The Decrease in HR in YGT-G

We found that HR decreased significantly by ~5 beats/min during fast and slow walking for the first and last sets of IWT after the 30th day of the training in the YGT-G, but not in CHO-G (Figure 3 and Figure 4, respectively).

Regarding the mechanisms for the decreases in HR in YGT-G, an increase in cardiac stroke volume with PV expansion might be involved. Okazaki et al. [6] examined the effects of post-exercise milk protein-CHO supplementation (12 g protein, 205 kcal) during an 8-week cycling training and reported a ~6% increase in PV compared, with the baseline values, by the supplementation, but no significant increase in PV by the CHO supplementation (0 g protein, 32 kcal). Moreover, they examined cardiovascular responses during 20-min of cycling exercise at the intensity of 60% of pre-training $\overset{\cdot }{V}$O_2peak_ at ~30 °C of T_a_ and ~50% of RH in an artificial chamber and suggested that cardiac stroke volume increased by ~10% with the milk protein-CHO supplementation, but not with the CHO supplementation. Similar results were obtained in our subsequent studies on both young [11] and older men [7]. Thus, the decreases in HRs after the 30th day of the training in YGT-G might have been caused by increased cardiac stroke volume with increased PV.

As for the mechanisms for PV expansion by milk protein-CHO intake, it was suggested in young subjects that the hepatic albumin synthesis rate was enhanced after a bout of intense exercise [19,20], and moreover, plasma protein synthesis in the liver was suggested to be enhanced after consuming, or following the intravenous infusion, of amino acids [21]. Furthermore, since it was suggested that insulin stimulates protein synthesis and suppresses proteolysis in the liver [22,23], the CHO contained in the supplement likely increased albumin synthesis when plasma amino acid concentrations were elevated by the supplement. When the albumin synthesized in the liver is released into plasma, it increases the plasma albumin concentration, enhances the effective colloid osmotic pressure gradient between intra- and extravascular spaces, and accelerates the water shift into the intravascular space to increase PV [24,25]. Experimentally, Okazaki et al. [26] suggested that the intake of a milk protein-CHO supplement immediately after a bout of intense exercise increased PV from the 1st hr in young subjects and the 2nd hr in older subjects, and that the increase was sustained until the 23rd hr after intake. These results suggest that the milk protein-CHO supplementation by yogurt intake immediately after daily IWT likely increased PV and cardiac stroke volume, resulting in a decrease in HR at a given intensity of walking.

### 5.3. Delayed Timing of the Decrease in HR in YGT-G

In the present study, the significant decrease in HR during IWT for YGT-G did not occur before the 30th day of the training, which was much longer than the 5–10 days required for the significant reduction in HR for young people in a laboratory-based study [11]. The detailed mechanisms for the delayed appearance of the effects of yogurt intake in older people have remained unknown; however, as one of the possible mechanisms, reduced glucose metabolism in older people might be involved. Uchida et al. [27] suggested that PV was lower in subjects with a higher fasting glucose concentration, and moreover, that milk protein-CHO intake during IWT for 5 months decreased hemoglobin A1c, which was closely associated with increases in plasma albumin content and PV; however, in their study, they did not measure HR during training. Recently, they measured blood glucose regulation for 24 h using a continuous glucose monitoring system (iPro2^R^, Medtronic, Minneapolis, MN, USA) before and after 5-mo IWT in two groups of mildly hyperglycemic older people, who consumed either milk protein-CHO supplementation or CHO-electrolyte solution supplementation during the training period [28]. They found that the blood glucose regulation was improved in the group with milk protein-CHO supplementation, but not in that with a CHO supplementation. These results suggest that glucose metabolism was involved in albumin synthesis in the liver, probably via insulin sensitivity, and thereby affecting PV expansion. The results in the present study suggest that it took more than 30 days to improve glucose metabolism enough to increase PV in older people.

### 5.4. ΔHR in YGT-G

As shown in Figure 5, we found no significant decrease in ΔHR with the constant ΔEI during the training in the YGT-G.

Okazaki et al. [6] measured esophageal temperature (T_es_) and HR during 20-min cycling exercise in older subjects in the conditions stated above, and suggested that HR increased rapidly from ~64 at rest to ~126 beats/min for the first 5 min of cycling exercise, but with no increase in T_es_ within the period, and then HR gradually increased by 12 beats/min as T_es_ increased from ~36.5 to ~37.8 °C. However, they suggested that the increases after 5 min in HR and T_es_ were reduced after aerobic training for 8 weeks with milk protein-CHO supplementation, while these reductions were minimal with placebo supplementation. These results suggest that the gradual increase in HR after the 5 min of exercise was caused by the increase of T_es_ through its direct stimulation to the pacemaker of the heart [16], and it was reduced by milk protein-CHO supplementation.

Regarding the greater reduction in the increase in T_es_ during exercise in a warm environment due to milk protein-CHO supplementation than due to CHO supplementation only, Okazaki et al. [6] suggested that sweat rate and cutaneous vasodilation responses to a given increase in T_es_ were increased by ~20% and ~70%, respectively, after training through milk protein-CHO supplementation, while they remained unchanged by CHO supplementation alone. As for the improved heat dissipation functions, these researchers suggested that the PV expansion with the supplementation increased the venous return to the heart, stimulated cardio-pulmonary receptors of the heart, and caused cutaneous vasodilation, which might improve sweat rate response by increasing skin blood flow to the sweat glands [6].

On the other hand, in the present study, we found no significant decrease in ΔHR during training in the YGT-G (Figure 5). The reason for the discrepancies between these two studies might be that the subjects in the present study did not rest long enough to decrease T_es_ to the baseline before starting IWT, so that thermoregulatory as well as HR responses to increased T_es_ might have already occurred for the first set of IWT, which might be quite different from the experimental condition in the previous laboratory-based studies, where subjects rested for ~60 min in an artificial climate chamber [6,7]. Indeed, we found that the HR during slow walking for the first set of IWT was ~90 beats/min in the CHO-G and the YGT-G (Figure 3), much higher than ~60 beats/min at rest before starting bicycling exercise in the previous studies [6,7], which might minimize the further increase in HR from the first to last set of IWT. As a result, ΔHR between the first and last sets of IWT during fast walking was only ~4 beats/min in both groups, ~30% of that from the first 5 min to 20 min of exercise in the previous studies [6,7]. Thus, the minimized ΔHR might make it difficult to distinguish the effect of the supplementation on improved thermoregulatory capacity from that on increased aerobic capacity. However, we believed that the HR at a given EI during outdoor IWT (Figure 3 and Figure 4) could be used as a combined index for aerobic and thermoregulatory capacities, which was improved after the 30th day of the training in YGT-G.

### 5.5. Limitations

In the present study, we used a yogurt drink for a supplement, since it contains amounts of milk protein-CHO similar to those used in the laboratory- and gym-based studies [6,7]. However, it contains greater amounts of electrolytes and a smaller amount of glucose compared with the supplement for the CHO-G (Table 4). Although we could not completely exclude their possible effects on the results, the differences in the amounts of electrolyte intake were negligible, considering the total amounts of their intake by diet (Table 5), and we previously confirmed that a smaller amount of glucose intake by milk protein-CHO supplementation than that by CHO supplementation had little effect on the results in the laboratory-based study [7]. Therefore, it is likely reasonable to explain the possible mechanisms for the reduction in HR in the YGT-G observed in the present study with reference to the results of previous studies [6,7]. Thus, the combined supplementations of milk protein and CHO immediately after daily exercise might be a key factor to obtain the noticed effects.

We used HR as a combined index of exercise and heat stress during IWT, without measuring changes in aerobic and thermoregulatory capacities, as was completed just before and after training in the laboratory- and gym-based studies [6,7], considering that the major aim of the present study was to examine any merits of the supplementation on time-series changes in tolerance to exercise and heat stress brought on by outdoor IWT. As a result, we found that HR decreased by ~5 beats/min after the 30th day of the training with no change in EI in the YGT-G, which was equivalent to an ~10% increase in the HR reserve for the subjects of this age. These results suggest the merits of milk protein-CHO supplementation during outdoor aerobic exercise to prevent heat illness and heat stroke in midsummer, as well to increase the time required for exhibiting these conditions.

### 5.6. Future Directions

In the present study, we found that yogurt intake reduced cardiovascular strain during outdoor walking in midsummer. Moreover, we previously reported that milk protein-CHO supplementation during IWT for five months enhanced the increases in thigh muscle mass and strength [29], along with enhanced proinflammatory gene suppression in the leukocytes [30] in middle-aged and older women. Furthermore, we reported that supplementation during IWT for five months improved glucose metabolism in middle-aged and older men and women, even in the intervention period comprising cool as well as hot seasons, from the middle of May to the end of October [28]. Finally, we reported that the supplementation during cycling training at ~25 °C for two months enhanced an increase in carotid arterial compliance in older men in the laboratory-based study [7]. These results suggest that yogurt intake during IWT improves the symptoms of age-associated diseases, with suppression of chronic inflammation of the body in middle-aged and older men and women, even if the protocol was implemented in cooler seasons.

In addition, before the present study, we could not find any previous intervention studies that documented how nutritional supplementation affects exercise training achievements and how the intervention affected health outcomes in a larger population of middle-aged and older people. This outcome might have been observed because it is more challenging to conduct facility- or field-based interventions while monitoring EI as well as HR continuously in large cohorts of older people. On the other hand, recent the improvement of IoT technology and field sensors has made it possible for physiologists to take an epidemiological approach by monitoring these variables at every training session throughout an intervention period in the field. Since the system we developed is available at a lower cost and with minimal personnel compared to the requirements of the previous laboratory- and gym-based studies, such advancements in technology expand the potential to verify any effects of nutritional supplements during training on age-associated diseases, as well as heat illness, in middle-aged and older people.

## 6. Conclusions

Post-exercise yogurt intake appears to reduce cardiovascular strain during outdoor interval walking training in older people.

## Figures and Tables

**Table 1:** Physical characteristics of subjects by group.

	CHO-G *n* = 39	YGT-G *n* = 37
Age, yr	73 ± 5	73 ± 5
Height, cm	157 ± 6	157 ± 6
Body mass, kg	57.3 ± 9.0	54.7 ± 10.1
$\overset{\cdot }{V}$O_2peak_, mL·kg^−1^·min^−1^	25.1 ± 5.3	26.5 ± 5.8
HR_peak_, beats/min	142 ± 20	143 ± 19

Values are means ±S.D. CHO-G, the carbohydrate intake group; YGT-G, the yogurt intake group; $\overset{\cdot }{V}$O_2peak_, peak oxygen consumption rate during a graded walking test; HR_peak_, peak heart rate at $\overset{\cdot }{V}$O_2peak_. There were no significant differences in any parameters between the groups (all, *p* > 0.2).

**Table 2:** Past and current health status of participants.

	CHO-G *n* = 39	YGT-G *n* = 37
Anamnesis, %		
Hypertension	12.8	21.6
Heart disease	0.0	8.1
Diabetes mellitus	2.6	2.7
Hyperlipidemia	10.3	5.4
Orthopedic diseases	2.6	8.1
Other diseases	7.7	10.8
Current drinkers, %	56.4	56.8
Current smokers, %	5.1	0.0
Current medications to influence, %		
Autonomic function	30.8	32.4
Blood lipids	35.9	10.8
Blood glucose	10.3	2.7

CHO-G, the carbohydrate intake group; YGT-G, the yogurt intake group. From answers to questionnaire given to subjects before participating in the study, we confirmed that there were no significant differences in any indices for health status between the groups (all, *p* > 0.1). Other diseases included stroke, renal and hepatic diseases, and cancers.

**Table 3:** Training achievements for CHO-G and YGT-G.

	CHO-G *n* = 39	YGT-G *n* = 37
Total walking days	47 ± 1	47 ± 1
Walking days per week	5.8 ± 0.1	5.8 ± 0.1
Fast walking		
Time, min/walking day	21 ± 1	21 ± 1
§ Energy expenditure, L O_2_/walking day	21.9 ± 1.6	20.6 ± 1.4
Slow walking		
Time, min/walking day	23 ± 2	23 ± 2
§ Energy expenditure, L O_2_/walking day	13.4 ± 0.9	13.4 ± 1.3

Values are means ±SE. CHO-G, the carbohydrate intake group; YGT-G, the yogurt intake group. § Resting oxygen consumption is not included. There were no significant differences in any parameters between the groups (all, *p* > 0.5).

**Table 4:** The composition of supplements.

	Jelly Drink (180 g/Dose)	Yogurt Drink (318 g/Dose)
Total energy, kcal	180	192
Major nutrients		
Protein, g	0	9.3
Carbohydrate, g	45 (mainly dextrin)	39 (glucose 8.7 g, lactose 8.4 g, and others)
Fat, g	0	0
Other nutrients		
Dietary fibers, g	0	0
Na, mg	43	117
K, mg	50	410
Ca, mg	47	318
Mg, mg	0.83	35
Fe, mg	0.05	0.3

The carbohydrate intake group (CHO-G) consumed 180 g of the jelly drink and the yogurt intake group (YGT-G) consumed 318 g of the yogurt drink within 30 min after daily interval walking training (IWT), respectively.

**Table 5:** The composition of dietary intake per day during the training period.

	CHO-G *n* = 39	YGT-G *n* = 37
Total energy, kcal	1801 ± 40	1744 ± 47
Major nutrients		
Protein, g	70.1 ± 1.7	68.2 ± 1.7
Carbohydrate, g	233 ± 6	227 ± 6
Fat, g	57.5 ± 1.8	56.6 ± 1.8
Other nutrients or foods		
Dairy product, g	180 ± 17	154 ± 14
Sugar, g	8.1 ± 0.8	9.0 ± 0.9
Dietary fibers, g	15.4 ± 0.5	15.7 ± 0.5
Na, mg	4087 ± 129	4121 ± 135
K, mg	2604 ± 88	2566 ± 80
Ca, mg	557 ± 24	533 ± 24
Mg, mg	281 ± 10	271 ± 8
Fe, mg	7.9 ± 0.3	8.1 ± 0.3

Values are means ±SE. CHO-G, the carbohydrate intake group; YGT-G, the yogurt intake group. The values do not include the compositions of the supplements. There were no significant differences in any components between the groups (all, *p* > 0.2).

## Data Availability

Data will be provided by the corresponding author upon request.

## Abbreviations

IWT, interval walking training; CHO-G, the carbohydrate intake group; YGT-G, the yogurt intake group; EI, exercise intensity; HR, heart rate; PV, plasma volume; IoT, Internet of things; $\overset{\cdot }{V}$O_2peak_, peak aerobic capacity; T_a_, atmospheric temperature; RH, relative humidity; ΔEI, a difference in EI between the first and last sets of IWT; ΔHR, a difference in HR between the first and last sets of IWT; SE, standard error.

## References

[1] Ministry of Environment Environmental Health Manual 2022. https://www.wbgt.env.go.jp/heatillness_manual.php.

[2] Ho C.W., Beard J.L., Farrell P.A., Minson C.T., Kenney W.L. (1997). Age, fitness, and regional blood flow during exercise in the heat. J. Appl. Physiol..

[3] Kenney W.L., Ho C.W. (1995). Age alters regional distribution of blood flow during moderate-intensity exercise. J. Appl. Physiol..

[4] Okazaki K., Kamijo Y., Takeno Y., Okumoto T., Masuki S., Nose H. (2002). Effects of exercise training on thermoregulatory responses and blood volume in older men. J. Appl. Physiol..

[5] Thomas C.M., Pierzga J.M., Kenney W.L. (1999). Aerobic training and cutaneous vasodilation in young and older men. J. Appl. Physiol..

[6] Okazaki K., Ichinose T., Mitono H., Chen M., Masuki S., Endoh H., Hayase H., Doi T., Nose H. (2009). Impact of protein and carbohydrate supplementation on plasma volume expansion and thermoregulatory adaptation by aerobic training in older men. J. Appl. Physiol..

[7] Kataoka Y., Kamijo Y.I., Ogawa Y., Sumiyoshi E., Nakae M., Ikegawa S., Manabe K., Morikawa M., Nagata M., Takasugi S. (2016). Effects of hypervolemia by protein and glucose supplementation during aerobic training on thermal and arterial pressure regulations in hypertensive older men. J. Appl. Physiol..

[8] Masuki S., Morikawa M., Nose H. (2020). Internet of Things (IoT) System and Field Sensors for Exercise Intensity Measurements. Compr. Physiol..

[9] Yamazaki T., Gen-No H., Kamijo Y., Okazaki K., Masuki S., Nose H. (2009). A new device to estimate VO2 during incline walking by accelerometry and barometry. Med. Sci. Sports Exerc..

[10] Masuki S., Morikawa M., Nose H. (2019). High-Intensity Walking Time Is a Key Determinant to Increase Physical Fitness and Improve Health Outcomes After Interval Walking Training in Middle-Aged and Older People. Mayo Clin. Proc..

[11] Goto M., Okazaki K., Kamijo Y., Ikegawa S., Masuki S., Miyagawa K., Nose H. (2010). Protein and carbohydrate supplementation during 5-day aerobic training enhanced plasma volume expansion and thermoregulatory adaptation in young men. J. Appl. Physiol..

[12] Yoshida Y., Sasaki G., Goto S., Yanagiya S., Takashina K. (1975). Studies on the etiology of milk intolerance in Japanese adults. Gastroenterol. Jpn..

[13] Kolars J.C., Levitt M.D., Aouji M., Savaiano D.A. (1984). Yogurt—An autodigesting source of lactose. N. Engl. J. Med..

[14] Morikawa M., Masuki S., Furuhata S., Shimodaira H., Furihata M., Nose H. (2018). Interval walking training over 10 years protects against age-associated declines in physical fitness. FASEB J..

[15] Nemoto K., Gen-no H., Masuki S., Okazaki K., Nose H. (2007). Effects of high-intensity interval walking training on physical fitness and blood pressure in middle-aged and older people. Mayo Clin. Proc..

[16] Gonzalez-Alonso J., Mora-Rodriguez R., Below P.R., Coyle E.F. (1995). Dehydration reduces cardiac output and increases systemic and cutaneous vascular resistance during exercise. J. Appl. Physiol..

[17] Dunn A.L., Marcus B.H., Kampert J.B., Garcia M.E., Kohl H.W., Blair S.N. (1999). Comparison of lifestyle and structured interventions to increase physical activity and cardiorespiratory fitness: A randomized trial. JAMA.

[18] Morikawa M., Okazaki K., Masuki S., Kamijo Y., Yamazaki T., Gen-no H., Nose H. (2011). Physical fitness and indices of lifestyle-related diseases before and after interval walking training in middle-aged and older males and females. Br. J. Sports Med..

[19] Nagashima K., Cline G.W., Mack G.W., Shulman G.I., Nadel E.R. (2000). Intense exercise stimulates albumin synthesis in the upright posture. J. Appl. Physiol..

[20] Yang R.C., Mack G.W., Wolfe R.R., Nadel E.R. (1998). Albumin synthesis after intense intermittent exercise in human subjects. J. Appl. Physiol..

[21] De Feo P., Horber F.F., Haymond M.W. (1992). Meal stimulation of albumin synthesis: A significant contributor to whole body protein synthesis in humans. Am. J. Physiol..

[22] Ahlman B., Charlton M., Fu A., Berg C., O’Brien P., Nair K.S. (2001). Insulin’s effect on synthesis rates of liver proteins. A swine model comparing various precursors of protein synthesis. Diabetes.

[23] De Feo P., Gaisano M.G., Haymond M.W. (1991). Differential effects of insulin deficiency on albumin and fibrinogen synthesis in humans. J. Clin. Investig..

[24] Convertino V.A., Brock P.J., Keil L.C., Bernauer E.M., Greenleaf J.E. (1980). Exercise training-induced hypervolemia: Role of plasma albumin, renin, and vasopressin. J. Appl. Physiol. Respir. Environ. Exerc. Physiol..

[25] Gillen C.M., Lee R., Mack G.W., Tomaselli C.M., Nishiyasu T., Nadel E.R. (1991). Plasma volume expansion in humans after a single intense exercise protocol. J. Appl. Physiol..

[26] Okazaki K., Hayase H., Ichinose T., Mitono H., Doi T., Nose H. (2009). Protein and carbohydrate supplementation after exercise increases plasma volume and albumin content in older and young men. J. Appl. Physiol..

[27] Uchida K., Kamijo Y.I., Ikegawa S., Hamada K., Masuki S., Nose H. (2018). Interval walking training and nutritional intake to increase plasma volume in elderly. Med. Sci. Sports Exerc..

[28] Uchida K., Masuki S., Morikawa M., Furihata M., Manabe K., Ogawa Y., Kataoka Y., Aida T., Nakano S., Nose H. (2018). Milk plus carbohydrate supplementation during interval walking training enhanced the improvement of blood glucose and blood pressure regulations in older people. FASEB J..

[29] Okazaki K., Yazawa D., Goto M., Kamijo Y.I., Furihata M., Gen-no H., Hamada K., Nose H. (2013). Effects of macronutrient intake on thigh muscle mass during home-based walking training in middle-aged and older women. Scand. J. Med. Sci. Sports.

[30] Masuki S., Nishida K., Hashimoto S., Morikawa M., Takasugi S., Nagata M., Taniguchi S., Rokutan K., Nose H. (2017). Effects of milk product intake on thigh muscle strength and NFKB gene methylation during home-based interval walking training in older women: A randomized, controlled pilot study. PLoS ONE.

